# Targeting MCT-1 oncogene inhibits Shc pathway and xenograft tumorigenicity

**DOI:** 10.18632/oncotarget.688

**Published:** 2012-11-09

**Authors:** Hung-Ju Shih, Hsiao-Huei Chen, Yen-An Chen, Meng-Hsun Wu, Gan-Guang Liou, Wei-Wen Chang, Linyi Chen, Lu-Hai Wang, Hsin-Ling Hsu

**Affiliations:** ^1^ Institute of Molecular and Genomic Medicine, National Health Research Institutes, Taiwan; ^2^ Division of General Surgery, Wan Fang Hospital, Taipei Medical University, Taiwan; ^3^ Institute of Molecular Medicine and Department of Medical Science, National Tsing Hua University, Taiwan

**Keywords:** MCT-1, Shc, Ras, ERK, apoptosis, tumor

## Abstract

Overexpression of Shc adaptor proteins is associated with mitogenesis, carcinogenesis and metastasis. Multiple copies in T-cell malignancy 1 (MCT-1) oncoprotein promotes cell proliferation, survival and tumorigenic effects. Our current data show that MCT-1 is a novel regulator of Shc-Ras-MEK-ERK signaling and MCT-1 is significantly co-activated with Shc gene in human carcinomas. The knockdown of MCT-1 enhances apoptotic cell death accompanied with the activation of caspases and cleavage of caspase substrates under environmental stress. The cancer cell proliferation, chemo-resistance and tumorigenic capacity are proved to be effectively suppressed by targeting MCT-1. Accordingly, an important linkage between MCT-1 oncogenicity and Shc pathway in tumor development has now been established. Promoting MCT-1 expression by gene hyperactivation may be recognized as a tumor marker and MCT-1 may serve as a molecular target of cancer therapy.

## INTRODUCTION

The development of cancer can be attributed to biological aberrations, including the ability to sustain proliferative signaling, to avoid growth suppressors, to resist cell death, to replicate indefinitely, to stimulate angiogenesis and chromosomal instability [1]. Src homolog and collagen homolog (Shc) (gene aliases, Shc 1 and ShcA), the adaptor proteins transmit the signaling of cell surface receptors [2], such as EGF receptor (EGFR) [3], erbB-2 receptor [4] and insulin receptor [5]. Shc function was proved to be sufficient for tumor progression in MMTV/MT transgenic mice by knock-in alleles of the ShcA gene [6]. ShcA adaptor also plays a critical role in TGF-β- and Neu/ErbB-2-induced breast cancer cell motility and invasion [7]. The abundance of Shc protein is concomitant with human endometriosis which is associated with ERK1/2 hyperactivation and susceptibility to breast cancer [8]. Shc proteins have three isoforms with distinct molecular weights (46, 52 and 66 kDa), playing multiple important roles in mitogenesis, carcinogenesis and metastasis [3, 9, 10]. For example, p52^Shc^ and p46^Shc^ activate Ras-ERK pathway [9, 12]. But, p66^Shc^ was originally recognized as an inhibitor of ERK1/2 activity and found to antagonize mitogenic and surviving abilities of T-lymphoma Jurkat cell line [13]. The increase in p66^Shc^ promoted the stress-induced apoptosis and the p66^Shc^ knockout mice were confirmed to have a longevity effect [11, 13, 14]. Moreover, p66^Shc^ restrains Ras hyper-activation, Rb-dependent proliferation and metastatic feature of lung carcinoma cells [15].

Paradoxically, p66^Shc^ activates ERK1/2 and enhances proliferation of prostate cancer cells [16]. As well as, abundant expression of p66^Shc^ was also recognized in many breast cancer cell lines and primary breast tumors with high metastatic potential [17], suggesting its important impact on tumorigenicity. Moreover, p66^Shc^ protein level is induced by steroid hormone in the proliferation of several carcinoma cells and in primary prostate cancers [18]. The novel roles of Shc proteins, especially p52^Shc^ and p66^Shc^, have been identified in steroid hormone-regulated cancers and metastasis [10]. p66^Shc^ is also functionally involved in regulating oxidative stress-induced apoptosis which mediates steroid action via the redox signaling pathway [19]. Signaling activation of Shc is thus implicated in tumorigenesis and the behaviors of cancer cells, suggesting Shc's potential as a prognosic marker and a target for cancer treatment.

Multiple Copies in T-cell malignancy 1 (MCT-1) also known as Malignant T Cell-amplified Sequence 1 (MCTS1) was originally identified in a lymphoma cell line [20]. Ectopic MCT-1 expression elicits CDK4/CDK6 kinase activity and Cyclin D1 accumulation, thereby stimulating cell proliferation [21]. The oncogenic MCT-1 enhances AKT activity and protects cells against programmed death under environmental stress [22]. In addition to be phosphorylated *in vitro* by CDC2 and involved in cell cycle progression [20, 23], MCT-1 protein physically interacts with the centrosomal apparatus and regulates mitotic progression and spindle assembly [24]. Overexpression of MCT-1 oncogene transforms NIH3T3 (murine fibroblasts) and MCF-10A (human breast epithelia) cells [20, 25]. Cells introducing MCT-1 evade growth suppression and checkpoint control as well as proficiently promote p53 destabilization via an ubiquitin-proteasome pathway following DNA damage [26]. The synergistic promotions on the cell migration and tumorigenic process have been demonstrated in MCT-1 overexpression alongside p53 deficiency [27, 28]. Intriguingly, induction of MCT-1 in the p53-deficient cells advances ERK1/2 activity [26], genomic instability [27], nuclear aberrations and mitotic catastrophes [24]. Furthermore, the posttranslational regulations associated with Hu Antigen R (HuR) which connects to the enhanced translation of tumor-promoting genes, such as Cyclin D1, or the decreased translation of tumor-suppressing genes, such as caspase 2, are altered by overexpressing MCT-1 [29]. Relating to the HuR function and promoting of the angiogenicity [30, 31], the angiogenesis inhibitor thrombospondin-1 (TSP-1) is suppressed by the induction of MCT-1.

We demonstrate for the first time that both MCT-1 and Shc genes are highly activated in human cancers. Targeted suppression of MCT-1 promotes caspase activation, apoptosis and chemo-sensitivity but inhibits Shc expression, anchorage-independent growth and xenograft tumorigenicity.

## RESULTS

### High expression of MCT-1 and Shc genes in human cancers

MCT-1 promotes angiogenicity and tumorigenicity in cancer cell xenografted mice [27, 28, 30]. The TissueScan Lung Cancer Tissue qPCR Array (Panel II, III and V) (OriGene Technologies, Inc.,) was analyzed the level of MCT-1 mRNA expressed in human lung carcinomas, in which the MCT-1 mRNA revealed a 2-fold induction over the mean of normal lung tissue were recognized as high expression of MCT-1 gene. Accordingly, MCT-1 gene was observed to be significantly induced in stage I (83.3%), stage II (76.7%), stage III (85.3%) and stage IV (100%) of 124 lung cancer patients (Table 1). Overall, 83.9% of the cancer samples showed a significant elevation of MCT-1 mRNA level, indicating the clinical relevance of MCT-1 gene stimulation in lung carcinomas. Shc induction is implicated in tumorigenesis [6, 10, 19]. As examined in Shc mRNA level, we found that Shc gene was highly activated in different stages of lung cancer (Table 2). Overall, 62.1% of the 124 lung cancer patients had a significant induction of Shc gene. The frequency of MCT-1 and Shc gene co-activation was again studied, and the results showed that 58.1% of the cancer patients exhibited high activation of both MCT-1 and Shc genes but only 11.3% of cases expressed low-level of both genes (Table 3). The data of positive association of Shc and MCT-1 gene activation in human lung cancers was statistically significant (p< 0.0001).

**Table 1:** MCT-1 mRNA expression levels in human lung cancers The TissueScan lung cancer tissue cDNA arrays Panel II, III and V consisted of a total of 19 normal lung samples and 124 lung cancer biopsies from different individuals were analyzed the expression of MCT-1 mRNA by Q-RT-PCR. The MCT-1 mRNA level in each tumor sample was normalized to β-actin mRNA and calibrated to the overall mean of MCT-1 mRNA level of normal tissue (set as 1-fold). MCT-1 mRNA had a >2-fold induction in tumor samples over normal lung tissue were defined as the gene high-activation. The statistical analysis used Fisher’s exact test.

stage	MCT-1 high	MCT-1 low	Total	p-value
**normal**	**0** **(0%)**	19 (100%)		
**I**	**40** **(83.3%)**	8 (16.7%)	48 (38.7%)	*p*< 0.0001
**II**	**23** **(76.7%)**	7 (23.3%)	30 (24.2%)	*p*< 0.0001
**III**	**29** **(85.3%)**	5 (14.7%)	34 (27.4%)	*p*< 0.0001
**IV**	**12** **(100%)**	0 (0%)	12 (9.7%)	*p*< 0.0001
**Total**	**104** **(83.9%)**	20 (16.1%)	124 (100%)	*P*<0.0001

**Table 2:** Shc mRNA expression levels in human lung cancers The TissueScan lung cancer tissue cDNA arrays (Panels II, III and V) were used to analyze the Shc gene (three isoforms) expression by Q-RT-PCR analysis. The Shc mRNA level identified in tumors was normalized to β-actin and calibrated to the overall mean of Shc mRNA level of normal lung tissue. Shc mRNA levels in tumors which elevated a 1.5- fold increase over normal breast tissues were defined as the high-activation of Shc gene. Shc transcripts were observed to be induced in lung cancers. The statistical analysis used Fisher’s exact test.

stage	Shc high	Shc low	Total	p-value
**normal**	**2** **(12.5%)**	14 (87.5%)		
**I/II**	**51** **(65.4%)**	27 (34.6%)	78 (62.9%)	*p*< 0.0001
**III/IV**	**26** **(56.5%)**	20 (43.5%)	46 (37.1%)	*P*= 0.003
**Total**	**77** **(62.1%)**	47 (37.9%)	124 (100%)	*P*=0.0002

The Breast Cancer Tissue qPCR Array (Panel III and IV) (OriGene Technologies, Inc.,) was further studied to explore the linkage of Shc and MCT-1 genes that highly induced in another type of human cancer. Among 92 breast cancer tumors, we found that 56.5% of the biopsies had dual activation of Shc and MCT-1 genes, but only 14.1% of the samples had low-expression in both genes (Supplementary Table 1). Highly concomitant activation of Shc and MCT-1 genes was also observed in human breast cancer (p<0.0001), revealing their clinical relevance on mammary tumorigenicity as well.

**Table 3:** Table 3: The association of MCT-1 and Shc gene activation in lung cancer patients (LCPs) The co-activation of MCT-1 and Shc genes among the 124 human lung cancers were studied. Overall, 58.1% of the LCPs highly expressed both MCT-1 and Shc genes, but only 11.3% of the biopsies exhibit low expression of both genes. The correlation of MCT-1 and Shc gene induction is statically significant (*p*< 0.0001). The statistical analysis used Fisher’s exact test.

	Shc (high)	Shc (low)
MCT-1 (high)	**72 (58.1%)**	32 (25.8%)
MCT-1 (low)	6 (4.8%)	**14 (11.3%)**

*p*< 0.0001 (high : low expression of both MCT-1 and Shc genes)

### MCT-1 regulates the signaling cascade of Shc-Ras-MEK-ERK

To investigate the role of MCT-1 in Shc signaling pathway, the short hairpin RNA (shRNA) were transfected into MCF-10A, H1299 and A549 cells to knockdown MCT-1 gene expression (Supplementary Fig. 1A-C). Based on the level of MCT-1 protein production, the vector control transfectant was named “High” (with a maximal-amount of MCT-1), and the MCT-1 shRNA transfectants were named “Medium” (with a middle-level of MCT-1) and “Low” (with a low-level of MCT-1). As evaluated by flow cytometry analysis in regular culture condition, no significant change in the cell cycle profiling was observed upon MCT-1 reduction (Supplementary Fig. 1D).

**Figure 1:**
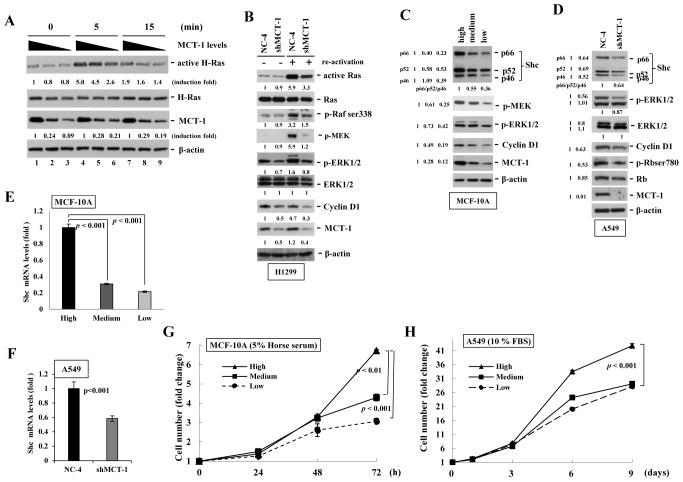
Knockdown of MCT-1 inhibits Shc-Ras-MEK-ERK signaling and cell proliferation (A) MCF-10A cells starved for 24 h were sub-cultured in regular media for different times. GTP-bound Ras protein was isolated from cell extracts by RBD domain-coupled sepharose. The level of active H-Ras was normalized with β-actin amount before comparing with the active H-Ras under the serum starvation (set as 1-fold). H-Ras activity is decreased by suppressing MCT-1. (B) H-Ras activity and its downstream signaling molecules were also inhibited in H1299 cells with MCT-1 knockdown. (C) Together with the reduction of Shc proteins, the accumulation of Cyclin D and the phosphorylation of MEK and ERK were all steadily suppressed by depleting MCT-1 in MCF-10A cells. (D) Abolition of MCT-1 in A549 cell also decreases the expression of Shc, p-ERK, Cyclin D1 and p-Rb than the control cells. (E-F) As analyzed by Q-RT-PCR, Shc mRNA level was observed to be much reduced corresponding to the degree of MCT-1 reduction independent of cell types. (G) MCF-10A cells were cultured in regular media for different periods followed by MTT assay. Significant growth attenuation was detected by 72 h in the absence of MCT-1. (H) A549 cells were cultured in regular media and cell proliferation rate was much reduced upon MCT-1 knockdown. The fold-change of each protein was quantified as compared to the vector control (set as 1-fold).

Shc participates in regulation of Ras signaling activation [2]. H-Ras activity was first examined to define whether the decrease in MCT-1 suppressed this essential component controlling cell proliferation (Fig. 1A). MCF-10A cells were starved for 24 h, followed by re-activation with serum for different time (5 and 15 min). The active H-Ras isolated by Raf-1 RBD-coupled sepharose was normalized to total H-Ras levels before comparing with the non-silenced control cells cultured in a serum-free condition (time 0). After stimulation for 5 min, H-Ras activity showed a 5-fold increase over the serum-depleted control cells with a high-level of MCT-1 (quantified as 1-fold). However, H-Ras activity was less induced when the MCT-1 knockdown cells were stimulated by the serum for 5 min (2.6-fold) and 15 min (1.4-fold) (lanes 6 and 9). Reduced H-Ras activity also was recognized in H1299 cells while depleting of MCT-1 (Fig. 1B). Consistent with inhibition of H-Ras activity, the phospho-activation of Raf, MEK and ERK1/2 were also found to be decreased upon MCT-1 reduction. Cyclin D1, a proliferation marker transcriptionally regulated by ERK [32, 33], was again observed to be consistently reduced by MCT-1 knockdown. Intriguingly, along with decrease in MEK-ERK signaling activation, the expression of Shc isoforms (p66^Shc^, p52 ^Shc^, and p46 ^Shc^) were dramatically suppressed corresponding to the degree of MCT-1 knockdown in MCF-10A cells (Fig. 1C). Similar results were identified in A549 cellular context that Shc proteins, Cyclin D1, p-ERK and p-Rb were all decreased by loss of MCT-1 (Fig. 1D). Further analysis of Shc mRNA level indicated that knockdown of MCT-1 inhibited Shc gene transcription, most likely due to MCT-1's impact on Shc gene transcription that accounted for the decrease of Shc protein (Fig. 1E and 1F).

The direct effect of MCT-1 knockdown on cell propagation was evaluated by MTT assay. After cultivation in regular media for 3 days, MCF-10A proliferation rate showed a significant difference comparable to the amounts of MCT-1 expression (Fig. 1G). MCT-1's role in regulating A549 cell proliferation was next studied in regular media (Fig. 1H). At day 9, the control A549 cells with a high-level of MCT-1 proliferated more rapidly (42-fold increase), than those of cells with a medium-level (29-fold increase) or with a low-level (28-fold increase) of MCT-1 protein. Thus, MCT-1 regulates cell proliferation attended with control of the Shc pathway.

### Knockdown of MCT-1 enhances spontaneous cell death and activates caspase 3

**Figure 2:**
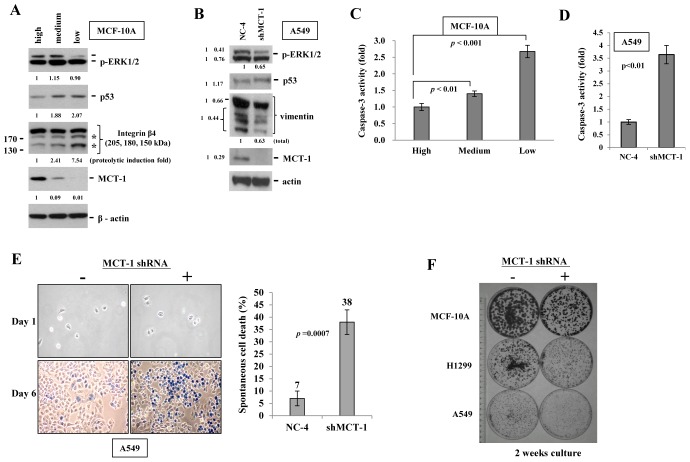
Spontaneous cell death induced by silencing of MCT-1 (A) MCF-10A cells were cultured in regular media for 5 days. Knockdown of MCT-1 decreases p-ERK expression but enhances p53 accumulation and integrin β4 cleavages (denoted by asterisks). (B) The levels of p-ERK and vimentin are reduced by suppressing MCT-1 in A549 cells as well. The changes in total amount or in the cleaved products were indicated. (C) MCF-10A cell extracts were incubated with the Ac-DEVD-pNA followed by the spectrophotometric detection of the chromophore *p*-nitroaniline (*p*-NA). Cells expressed a medium-level or a low-level of MCT-1 reveal higher caspase 3 activity than the control cells with a high-level of MCT-1. (D) Inhibition of caspase 3 activity was observed in A549 cells with MCT-1 reduction. (E) Trypan blue exclusion assay analyzed A549 cell viability upon regular cultivation for 5 days. Spontaneous cell death (in blue) was induced in MCT-1 depletion. (F) Cells were cultured at a low density (1000 cells/100 mm dish) in the respective regular media for 2 weeks. Clonogenic growth was detected by crystal violet staining. Colony number and growth are decreased upon MCT-1 knockdown.

ERK activates MDM2 that promotes p53 degradation [34]. We here identified that knockdown of MCT-1 in MCF-10A cells led to ERK dephosphorylation and p53 accumulation as cultured in regular media for 5 days (Fig. 2A). Active caspases trigger programmed cell death [35]. Integrin *β* 4 and vimentin are cleaved by caspase 3 and 7 in apoptosis, which have been recognized as potential molecular targets for cancer treatment [36-38]. The proteolysis of integrin *β* 4 (indicated by asterisks) and the accumulation of p53 were positively correlated with the decrease of MCT-1 in MCF-10A cells (Fig. 2A). Similarly, the levels of vimentin and p53 presentation were decreased by suppressing MCT-1 in A549 cells (Fig. 2B). Caspase 3 activity was again analyzed by the cleavage of colorimetric peptide Ac-DEVD-*p*NA and the release of chromophore *p*-nitroaniline (*p*NA) (Fig. 2C). The quantitative data revealed more than a 1.5-fold and a 2.5-fold increase in the relative activity of caspase 3 as MCT-1 differentially reduced in MCF-10A cells (Medium and Low). Inhibition of caspase 3 activity was also consistently identified in A549 cells lack of MCT-1 (shMCT-1) (Fig. 2D). Caspase 3 activation and p53 accumulation enhance apoptotic effects [39, 40]. To exam whether intrinsic apoptosis was induced by targeted suppression of MCT-1, A549 cell viability were determined by exclusion trypan blue effect as cells cultured in normal media for 5 days. The apoptotic rate was found to be much enhanced in knockdown of MCT-1 (38%) even in basal, unstressed culture condition, comparing to high survival ability of the comparable control cells (7%) (Fig. 2E). In consistence, MCF-10A cell viability was affected by the degree of MCT-1 reduction in regular culture (Supplementary Fig. 1E, indicated by arrowheads). We next evaluated cell expansion ability at a very low cell density (1000 cells/100 mm dish) as described previously [41]. After incubation in regular media for 2 weeks (Fig. 2F), the clonogenic cell growth were evaluated by crystal violet staining and the results identified a significant decrease of clonogenic capability upon knockdown of MCT-1 in different cell types.

### The enhanced cleavage of integrin β4, PARP and pro-caspases after MCT-1 reduction

**Figure 3:**
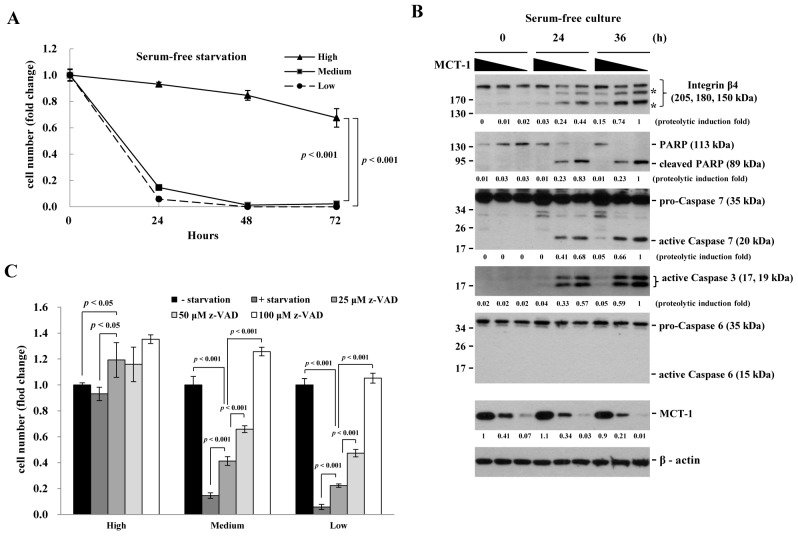
Loss of MCT-1 enhances apoptotic rate under environmental stress (A) MCF-10A cells cultured in the serum-free media were evaluated by MTT analysis at the indicated time. The MCT-1 knockdown cells (Medium and Low) exhibit low survival rates compared to the control cells with a high-level of MCT-1. (B) The proteolysis of integrin β4 and PARP proteins (denoted by asterisks) are synergistically enhanced by MCT-1 reduction and serum depletion. The pro-caspase 3 and 7 but not 6 are cleaved to be active, particularly in MCT-1 deficient condition. Each proteolytic product or active caspase was quantified. (C) Different concentrations of z-VAD-FMK were added to the serum-free media for 24 h in MCF-10A culture. z-VAD-FMK prevents apoptosis caused by starvation and MCT-1 deficiency in a dose-dependent manner.

To again exam if the loss of MCT-1 promoted apoptosis under the starvation condition, MCF-10A cells were starved for the indicated time (Fig. 3A). Being in the serum-free environment for 24 h, apoptotic rates were greatly promoted in cells with a medium-level (85%) or with a low-level (94%) of MCT-1 than that of control cells (7%). Upon fasting for 72 h, most of the MCT-1-deficient cells entered apoptotic death, but only 20% of control cells died from such stress.

Poly (ADP-ribose) polymerase (PARP) is cleaved by active caspases in apoptosis [42]. Integrin *β*4 (205 kDa) is cleaved into 180- and 150-kDa products during apoptosis by caspase 3 and 7 [38]. During 24 h and 36 h starvation, not only the integrin β4 but also the PARP were increasingly cleaved due to MCT-1 reduction (Fig. 3B). Since the specific proteolytic products of integrin *β*4 (180 and 150 kDa) (indicated by asterisks) were steadily accumulated in a fasting time-dependent and in a MCT-1 dose-dependent manner, thereby we speculated that the cleavage of integrin *β*4 relied more on the activity of caspases 3 and 7 in the MCT-1 knockdown cells. Consistent with this notion, the proteolytic activation of caspases 3 and 7 but not caspase 6 were detected (panels 3-5), showing that the caspase cascade was enhanced upon decrease of MCT-1.

To study whether the caspase activation mediated apoptosis upon MCT-1 reduction (Fig. 3C), MCF-10A cells were cultured in the serum-free media with different concentrations of a general caspase inhibitor, z-VAD-FMK (Caspase Inhibitor VI) [43]. By 24 h starvation, cell growth was decreased dramatically corresponding to the decrease of MCT-1 expression. In treatment of z-VAD-FMK (25 and 50 μM), the MCT-1 knockdown cells were protected from the apoptotic effect in a dose-dependent manner. As exposed to z-VAD-FMK at concentration of 100 μM, cell survival rates were restored even better than the un-stress and MCT-1-proficient state, indicating that the activation of caspases mediate the apoptotic effect collectively promoted by MCT-1 depletion and serum starvation.

### Inhibiting cancer cell growth and chemo-resistance by reduction of MCT-1

**Figure 4:**
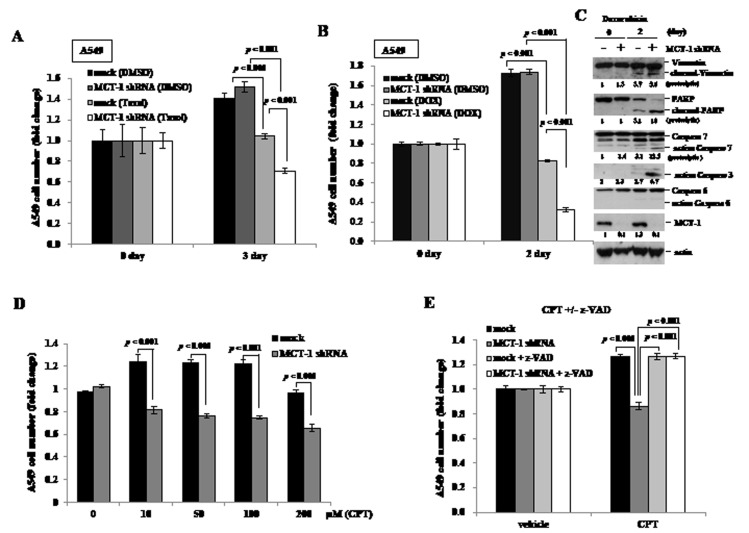
Loss of MCT-1 promotes chemo-sensitivity (A) A549 cells (2×10^4^/well) were treated with Taxol (0.1 μM) for 3 days and highly induced apoptotic effect in the MCT-1 shRNA transfectants. (B) Upon doxorubicin (DOX) (1 μM) treatment for 2 days, more A549 cell death was detected in the MCT-1-silencing condition. (C) Exposure to DOX induced more cleavage of vimentin and PARP as well as more active caspase 7 and caspase 3, especially in the MCT-1 knockdown cells. (D) Different concentrations of Camptothecin (CPT) were administrated with A549 cells for 24 h. MCT-1 shRNA transfection confers higher cellular sensitivity to CPT than the control cells (mock). (E) A549 cells were treated with 100 μM CPT alone or together with 50 μM z-VAD-FMK (z-VAD) for 24 h. The combination of CPT exposure and MCT-1 knockdown is inhibited by z-VAD that restores cell viability to the comparable control level.

We next investigated whether inhibition of MCT-1 conferred chemo-sensitivity of A549 cancer cells. Taxol, a mitotic inhibitor of cancer therapy [44], was treated cells for 3 days and the MCT-1 knockdown cells (MCT-1 shRNA) exhibited a higher susceptibility to Taxol that the apoptotic effect was much increased compared to the control cells (mock) (Fig. 4A). In the response to topoisomerase II inhibitor [45], Doxorubicin (DOX), for 2 days (Fig. 4B), A549 cell viability was found to be much reduced upon loss of MCT-1. Consistent with increased cytotoxicity, the cleavage of Vimentin and PARP, and the proteolytically-active caspase 7 and 3 were actually promoted by DOX treatment, particularly in combination with MCT-1 knockdown (Fig. 4C). Camptothecin (CPT), an inhibitor of topoisomerase I [46], its analogues are widely used in cancer chemotherapy [47, 48]. The MCT-1 knockdown cells also showed to be more susceptible to CPT treatment for 24 h at different concentrations (Fig. 4D), demonstrating a much higher CPT sensitivity than the vector control cells (mock). Inversely, the control cells showed a 20% increase in growth upon CPT administration (10-100 μM), and no considerable CPT susceptibility was noticed even upon exposure to 200 μM of CPT. To further exam whether the caspase activation involved in CPT-inhibitory effect, A549 cells were concomitantly treated with 100 μM CPT and 50 μM z-VAD-FMK for 24 h (Fig. 4E). The results confirmed that the inhibition of caspase activity prevented the MCT-1-depleted cells from cytotoxicity caused by CPT (MCT-1 shRNA+z-VAD). In contrast to 14% decrease in A549 cell viability in the absence of MCT-1, the unexpected 27% increase in control cell growth was identified upon CPT exposure for 24 h. Overall, the growth inhibition (41%) exhibited as a synergistic result of CPT administration and MCT-1 knockdown, was effectively abolished by z-VAD-FMK. Thereby, targeting MCT-1 by gene silencing sensitizes A549 cells to be inhibited by different chemo-agents.

### Knockdown of MCT-1 attenuates A549 tumorigenicity of xenograft mice

**Figure 5:**
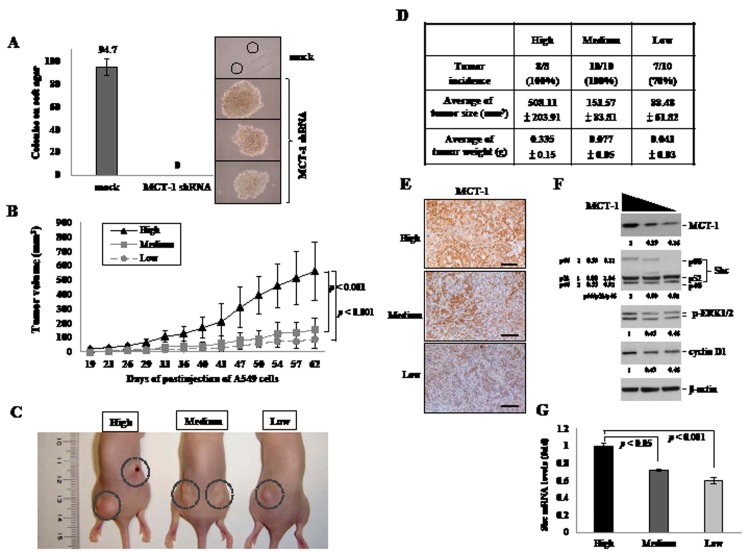
Targeted suppression of MCT-1 attenuates A549 tumor growth (A) A549 cells lose transformation ability after MCT-1 depletion as studied with soft agar assay. (B) A549 cells (1×10^6^ cells/site) were injected subcutaneously into athymic nude mice (n=10) on both flank regions. The tumor volumes were measured at the indicated time. Tumor development is much reduced corresponding to the degree of MCT-1 knockdown. (C) After injection for 2 months, smaller A549 tumors (enclosed in dotted circle) were emerged from the MCT-1 knockdown cells. (D) The quantification results indicate decreases in the tumor incidence, volume and mass after MCT-1 knockdown. (E) Immunohistochemistry identified a lower MCT-1 amount (brownish) in the tumor developed from the MCT-1 knockdown cells. Scale bar, 100 μm. (F) MCT-1 reduction suppresses the expression of p66^Shc^, Cyclin D1 and p-ERK in the tumors. The fold-change in total or each protein was indicated. (G) Shc transcripts are significantly decreased in the tumors derived from the MCT-1-deficient cells.

To study whether knockdown of MCT-1 inhibits malignant transformation (Fig. 5A), A549 lung cancer cells were performed the soft agar assay. The results showed that the cells silencing MCT-1 failed to grow in a viscous agarose and lost anchorage-independent growth. The phenotypic changes are assumed to be closely related to abort carcinogenic ability. To investigate the effect of MCT-1 knockdown on tumorigenicity, the A549 cells with differential level of MCT-1 protein were inoculated subcutaneously into BALB/c nude mice. The xenograft mice were maintained for 2 months, during which time tumor volumes were, measured every 3 or 4 days (Fig. 5B). The A549 cells present a high-level of MCT-1 yielded much larger tumors, but the tumor growth was apparently suppressed as MCT-1 reduced to either a medium or to a low level (Fig. 5C). The quantification data indicated that the tumor incidences decreased to 70% in A549 cells with a low-level of MCT-1 protein (Fig. 5D). The average tumor volume was also dramatically decreased corresponding to the extent of MCT-1 reduction from 508.11 mm^3^ in the control cells (high MCT-1) to 153.57 mm^3^ (medium MCT-1) and to 88.48 mm^3^ (low MCT-1), which also referred in the tumor weights (Fig. 5D).

As shown in immunohistochemistry, MCT-1 protein amounts contained in the A549 tumor decreased progressively matching to the degree of MCT-1 knockdown (Fig. 5E). Furthermore, the levels of p66^Shc^, Cyclin D1 and p-ERK in tumors were all comparatively reduced as MCT-1 was deficient (Fig. 5F). Downregulation of Shc mRNA levels in A549 tumors also reasonably explained the degree of Shc protein reduction (Fig. 5G), similar to that in the A549 cells (Fig. 1F). These results for the first time demonstrate the molecular linkage between MCT-1 expression and Shc signaling in tumor growth.

### Loss of MCT-1 suppresses H1299 xenograft tumorigenicity

**Figure 6:**
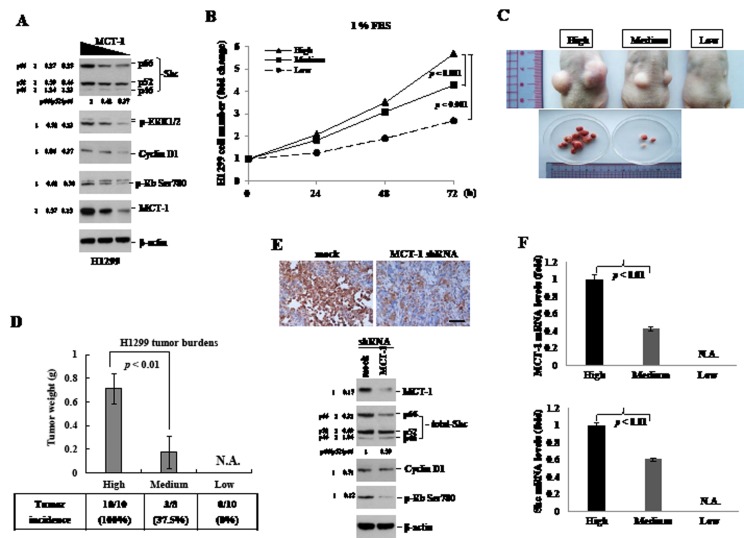
Decrease of MCT-1 protein suppresses H1299 xenograft tumorigenicity (A) The levels of Shc (p66/p52), Cyclin D1, p-ERK and p-Rb were found to be reduced by knockdown of MCT-1 in H1299 cells. The quantification results indicate the degree of individual or total protein reduction. (B) H1299 cells (5,000 cells/well) were cultured in the medium containing 1% FBS for different intervals. Cells deficient of MCT-1 (Medium and Low) grow much slower than the control cells with a high-level of MCT-1 (High). (C) BALB/c nude mice were injected subcutaneously with H1299 cancer cells (2×10^6^ cells/site) on both flank regions. Tumor growth is decreased dramatically due to MCT-1 depletion. (D) The quantification data show significant decreases of the tumor incidence and tumor mass derived from MCT-1-deficient cells (Medium and Low). (E) Immunohistochemistry reveals a high-abundance of MCT-1 in tumor came from the control cells (mock) than developed from MCT-1 knockdown cells (MCT-1 shRNA). Scale bar, 50 μm. Decreased expression in Shc (p66/p52), Cyclin D1 and p-Rb proteins were observed in the tumor with MCT-1 knockdown. The change in total or in individual protein was quantified. (F) Q-RT-PCR study quantified MCT-1 and Shc mRNA levels being reduced in the tumors with MCT-1 knockdown.

The targeted-inhibition of MCT-1 was again studied in H1299 cells. We found that the levels of p-ERK1/2, p-Rb, Shc (p66^/^p52) and Cyclin D proteins were consistently decreased after MCT-1 depletion (Fig. 6A). By culturing H1299 cells in 1% FBS medium for different periods (Fig. 6B), the control group displayed a 5.7-fold increase in cell number by 72 h, whereas the cells with a medium-level and a low-level of MCT-1 grew much slowly that exhibited a 4.3-fold and a 2.7-fold increase in cell number, respectively.

To further investigate tumor development in the deficiency of MCT-1, BALB/c nude mice were inoculated subcutaneously (s.c.) with H1299 cancer cells (2×10^6^) expressed various amounts of MCT-1 protein (High, Medium and Low) (Fig. 6C). A month after injection, tumor burdens were observed to be greatly reduced in the absent of MCT-1. At the time of sacrifice, no indication of tumor development from the H1299 cells with only a low-level of MCT-1 protein. The representative tumor images demonstrated a dramatic reduction of tumor loads upon MCT-1 inhibition. The statistically significant differences were detected in tumor incidences and tumor weights accounting to the levels of MCT-1 expression (Fig. 6D). Further examining the protein presented in the tumors, MCT-1, Shc (p66/p52), Cyclin D1 and p-Rb showed particular decreases in the context of MCT-1 depletion (MCT-1 shRNA) compared to the control cells (mock) (Fig. 6E). In agreements, MCT-1 and Shc mRNA levels were decreased by 40% and by 60%, coordinating with the status of MCT-1 reduced in tumors (Fig. 6F). Taken together, the attenuation or prevention of tumor growth because of targeting MCT-1 again closely connects to the inhibitory effect on Shc-ERK-Cyclin D1 cascade. Upregulation of Shc activity under MCT-1 oncogenic stress may potentially result in promoting cancer cell proliferation and tumor progression.

## DISCUSSION

### Knockdown of MCT-1 suppresses Shc signaling pathway

Our current results verify that Shc isoforms are differentially regulated by MCT-1 which directly affects the signaling cascade of Shc-Ras-ERK. The most significant effect occurs for the p66^Shc^ expression examined so far, presumably because of the unique tumorigenic role of p66^Shc^ in relation with MCT-1 oncogenic activity. Direct impact on Shc signaling cascade explains why gain-of-function MCT-1 promotes the proliferative rate of cells [20], and induces Cyclin D1 accumulation and Rb phosphorylation [21]. By supporting the xenograft tumorigenicity data (Fig. 5 and 6), the clinical information have first established the physiological relation between MCT-1 and Shc in human cancer development (Table 1-3 and supplementary Table 1).

Loss-of-function MCT-1 decreases Shc protein levels that presumably work through either by epigenetic modification or by post-transcriptional modulation involving MCT-1 activity. In the former case, Shc promoter function may be de-activated by targeting MCT-1, similar to inhibition of p53 gene promoter by overexpressing MCT-1 [28]. Shc promoter region has the putative Sp1 binding sites [49]. The Sp1-dependent gene transcription can be suppressed by the functional p53 [50]. MCT-1 increases Sp1 expression in the nucleus [25] and suppresses p53 mRNA homeostasis and p53 protein stability [26-28]. Therefore, the p53-mediated Sp1 reduction may be inversely elicited after MCT-1 depletion, by which attributes to Shc gene inhibition (Fig. 1E and 1F). The association of MCT-1 with ribosome may be another potential molecular mechanism in regulating the expression profiling of many signaling pathways [51], such as the Shc-Ras-MEK-ERK cascade. In the other way, MCT-1's function in promoting the efficient eIF2-independent recruitment of aminoacylated initiator tRNA (Met-tRNA^MET^_i_) to initiate translation machineray may be also involved in modulating Shc protein translation [52].

### Reduction of MCT-1 increases apoptotic event

Programmed cell death is a natural defender against cancer development [53], and malfunction of the apoptotic process can stimulate tumorigenesis [54]. Tumor cells have a tendency to be more resistant to environmental stress or chemotherapeutics partly due to the inactivation of pro-apoptotic signaling or the activation of anti-apoptotic pathway. In addition to prevent genomic instability and tumor development [55], the accumulation of p53 transcriptionally activates many downstream genes which stimulate the proteolytic activation of caspases to trigger cell death [40]. During apoptosis, PARP and integrin β4 are cleaved by the active caspases [38, 42]. Aberrant induction of integrin β4, stimulates malignant tumor progression and angiogenesis [56, 57]. Thus, targeting integrin β4 has been predicted as a rational approach for cancer and anti-angiogenic therapies [58]. The stimulus of p53 accumulation induces the activation of caspase 3 and 7, through which can promote apoptosis and enhance degradation of integrin β4 and PARP in targeted suppression of MCT-1 (Fig. 2 and 3). Accordingly, the loss-of-function MCT-1 raises the chemo-sensitivity of cancer cells (Fig. 4), implying that targeting MCT-1 may enhance the therapeutic efficacy on advanced and metastatic cancer.

### The association of MCT-1 and Shc gene activation in human cancers

Shc proteins couple the receptor tyrosine kinases (RTKs) to Ras signaling activation through association with Grb2 [59, 60]. Disrupting the interaction of Shc and Grb2 inhibits tumor growth of the xenograft mice [61], telling that Shc pathway may be served as an ideal cancer therapeutic target. Consistent with the results *in vitro* (Fig. 1C and 1D), significant inhibition of Shc expression in tumorigenic process are identified after MCT-1 depletion (Fig. 5 and 6), confirming that MCT-1 is a novel regulator of the Shc pathway. Thereby, blockade of MCT-1 activity potentially inhibits not only Shc signaling cascade but also the oncogenicity and tumorigenicity involving aberrant Shc activity. Clinical results also show important correlation between high-activation of both MCT-1 and Shc genes in different types of tumors. Given that the physiological connections and the clinical relevance of MCT-1 and Shc expression in development of tumor, understanding their molecular interaction may help to effectively prevent cancer cell propagation and tumor progression. The advanced investigation of MCT-1-Shc pathway that tumors are dependent upon for growth may facilitate the identification of new and effective therapeutics.

## METHODS

### Antibodies

Antibodies (Abs) specific for the cleaved caspase 3 (Asp175), caspase 6, caspase 7, Cyclin D1 (92G2), phospho-ERK1/2 (Thr202/Tyr204), ERK1/2, phospho-MEK1/2 (Ser217/221), MEK1/2, phospho-Rb (Ser780), Rb (4H1) and Shc were purchased from Cell Signaling Technology (Danvers, MA). Ha-Ras (MC57) and vimentin (V9) Abs were obtained from Millipore Corporation (Billerica, MA); Abs for integrin β4 and PARP were purchased from BD Biosciences (Franklin Lakes, NJ); and Abs for p53 (DO-1) and β-actin (AC-15) were bought from Santa Cruz Biotechnology (Santa Cruz, CA) and Novus Biologicals (Littleton, CO), respectively.

### Cell culture and transfection

Non-tumorigenic human mammary MCF-10A cells were cultured in DMEM/F-12 medium (GIBCO, Grand Island, NY) supplemented with 5% horse serum (HS) (GIBCO), 20 ng/ml EGF (ProSpec-Tany TechnoGene Ltd., Rehovot, Israel), 10 μg/ml insulin (Sigma, St. Louis, MO), 0.5 μg/ml hydrocortisone (Calbiochem, Darmstadt, Germany), 100 ng/ml cholera toxin (Sigma), 100 units/ml penicillin (GIBCO) and 100 μg/ml streptomycin (GIBCO) in a 5% CO_2_ incubator at 37°C. Non-small cell human lung carcinoma cell lines, H1299 and A549, were maintained in RPMI 1640 medium (GIBCO) supplemented with 10% heat-inactivated fetal bovine serum (FBS) (GIBCO), 2 mM/ml L-glutamine (GIBCO), 100 units/ml penicillin and 100 μg/ml streptomycin in a 5% CO_2_ incubator at 37°C. The pGeneClip MCTS1 shRNA (SA Biosciences Corp, Frederick, MD) or the control shRNA (mock) were transfected into cells using the jetPEI™ transfection reagent (Polyplus-transfection, New York, NY), followed by incubating with 0.5 μg/ml puromycin to select the line with differential reduction of MCT-1.

### Western blotting assay

CytoBusterTM Protein Extraction Reagent (Novagen, Darmstadt, Germany) including Protease Inhibitor Cocktail (Sigma) and Phosphatase Inhibitor Cocktail 2 (Sigma) was incubated with cells at 4°C for 30 minutes (min). Cell extracts were clarified by centrifugation at 16,000 rpm for 15 min at 4°C, and protein concentrations were determined by the Coomassie Plus™ Protein Assay Reagent (Thermo, Rockford, IL). The protein samples (15 μg) were mixed with TRICINE Sample Buffer (Protech Technology, Taipei, Taiwan) and heated at 95°C for 7.5 min. The samples were resolved on a 4–12% Bis/Tris NuPAGE gel (Invitrogen, Carlsbad, CA) and then transferred to a Hybond-C Extra membrane (Amersham Biosciences, Piscataway, NJ) using the Trans-Blot SD Semi-Dry Electrophoretic Transfer Cell (Bio-Red Laboratories, Hercules, CA). The membranes were blocked for 1 hour (h) in PBS with 5% Difco™ Skim Milk (BD Biosciences), hybridized with the indicated Abs and reactivated with horseradish peroxidase (HRP)-conjugated sheep anti-mouse IgG or donkey anti-rabbit IgG (Amersham Biosciences) before detecting with the SuperSignal^®^ West Pico Chemiluminescent Substrate (Thermo). The immune-reactive signals were stripped by Restore™ Western Blot Stripping Buffer (Thermo) before re-probing the protein of interest.

### Cell proliferation and viability

Cell proliferative and survival rates were measured by Cell Proliferation Kit I (Roche Diagnostics Corp., Basel, Switzerland). MCF-10A, H1299 and A549 cells were seeded in a BD Falcon™ 96-well microplate (5,000 cells per well) in triplicate and cultured in DMEM/F-12 complete medium with 5% HS or RPMI 1640 medium with 10% FBS as indicated. At the specific time, 10 μl of 3-(4,5-dimethylthiazol-2-yl)-2,5-diphenyltetrazolium bromide (MTT)-labeling reagent was added to each well for 4 h. The formazan crystals were dissolved with 100 μl of Solubilization solution and incubated overnight at 37°C before analyzing by the Infinite M200 Pro spectrophotometer (Tecan Group Ltd., Männedorf, Switzerland) at an absorbance of 595 nm.

Cell viability was analyzed by MTT assay. MCF-10A cells were seeded at 1×10^4^ cells per well and incubated with the serum-free DMEM/F-12 medium and different concentrations of Z-VAD for the indicated time. To test cellular sensitivity to chemotherapeutic agents, A549 cells were cultured at 2×10^4^ cells per well with 10% FCS containing RPMI 1640 medium and treated with 0.1 μM Taxol (Sigma), 1 μM Doxorubicin (DOX) (Sigma), or different concentrations of Camptothecin (CPT) (Sigma) for the indicated time.

### Cell cycle profiling and programmed cell death

To analyze the cell cycle distribution, cells were harvested by 0.25% trypsin/1 mM EDTA, washed with PBS, fixed in 70% ethanol and stored at −20°C. The cells were washed twice with ice-cold PBS and incubated with 10 μg/ml propidium iodide (PI) (Sigma) and 10 μg/ml RNase A in PBS (Sigma) at 4°C overnight. All the suspension and adhesion cells were collected, washed twice with PBS and stained with Annexin V apoptosis detection kit (BD Bioscience) for 15 min at room temperature. The Annexin V-positive cells were analyzed by the BD FACSCalibur flow cytometer (Becton-Dickinson, San Jose, CA). Both PI and Annexin V-FITC were excited at 488 nm, and the emissions were determined by FL2 PMT (564-606 nm bandpass filter) and FL1 PMT (515-545 nm bandpass filter). The results were analyzed using the Windows Multiple Document Interface (WinMDI), version 2.8 software developed by Scripps Research Institute (La Jolla, CA).

### Caspase 3 activity

Caspase 3 Colorimetric Activity Assay Kit (Millipore Corporation) was used to measure activity of caspase 3 by conversion of a colorimetric substrate, Ac-DEVD-*p*NA. The cells were cultured for 5 days and harvested by the Cell Lysis Buffer. The protein samples (100 μg) were incubated with 5X Assay Buffer, 10 μl Ac-DEVD-pNA and ddH_2_O in a total volume of 100 μl at 37°C for 2 h. The spectrophotometric detection of the chromophore *p*-nitroaniline (*p*-NA) was analyzed by the Infinite^®^ M200 Pro spectrophotometer at an absorbance of 405 nm.

### Ras activity assay

Cells were starved in the serum-free media for 24 h before serum activation for the indicated time. The active Ras was isolated by the agarose-coupled Ras-binding domain (RBD) of Raf-1 using the Ras Assay Reagent (Millipore Corporation). The cells were extracted in Magnesium-containing Lysis Buffer (MLB), 500 μg protein samples were incubated with 10 μg Raf-1 RBD-coupled agarose in a total volume of 500 μl and then gently rotated at 4°C for 1 h. Raf-1 RBD-coupled beads were collected by quick centrifugation at 14,000 g at 4°C and washed 3 times with ice-cold MLB. The active Ras bound to the Raf-1 RBD beads were resolved by SDS-PAGE and detected by H-Ras Ab.

### Trypan blue exclusion, clonogenic growth and anchorage-independent growth assays

Cells were cultured for 1 and 5 days in regular media and then stained with 0.4% trypan blue in PBS for 10 min. Trypan blue-positive cells indicating the dead cells without intact membrane were observed under a Nikon ECLIPSE TS100 inverted microscope and photographed by a Canon EOS 50D camera.

Cells were cultured at a low density (1000 cells/100 mm dish) in regular media for 2 weeks, the surviving clonogenic cells were washed by PBS, fixed with 50% and 100% methanol for 10 min each before staining with 0.05% crystal violet in 2% ethanol for 30 min. The clonogenic cells were photographed by a Canon Digital IXUS 850 IS camera.

A549 cells were performed soft agar assay to analyze the effect of anchorage-independent growth upon MCT-1 knockdown as described [25].

### Quantitative real-time polymerase chain reaction (Q-RT-PCR)

Total cellular RNA was isolated by TRIzol reagent (Invitrogen). The quality and quantity of RNA were determined by the NanoDrop ND-1000 Spectrophotometer (Thermo). Total RNA was reversely-transcribed into complementary DNA (cDNA) using the QuantiTect Reverse Transcription Kit (QIAGEN, Hilden, Germany). The specific probes (FAM dye-labeled MGB quencher) for MCT-1 (assay ID: Hs00273873_m1), Shc (assay ID: Hs01050691_g1) and beta-actin (assay ID: Hs99999903_m1) were purchased from Applied Biosystems (Forster City, CA). Q-RT-PCR was performed in triplicate in a 10 μl mixture, containing 4 ng cDNA, 0.5 μl TaqMan probe, 5 μl TaqMan PCR Master Mix (Applied Biosystems) and 2.5 μl deionized water (DNase-free and RNase-free). By use of ABI Prism 7900 Fast Real-Time PCR system (Applied Biosystems), PCR reactions were conducted as follows: 95°C for 10 min, 45 cycles of denaturing at 95°C for 15 seconds and annealing at 60°C for 1 min. The mRNA level of the target gene was normalized to β-actin mRNA level and calculated by the formula: Cycle threshold (ΔC_T_) = C_t_ target gene – C_T_ internal control. The relative mRNA level of each gene was determined by the formula: ΔΔC_T_ =ΔC_T_ control group – C_t_ experimental group. The fold change was calculated by the formula: 2^−ΔΔCT^.

### Tumorigenicity of cancer cell xenograft mice

To assess the tumorigenic effect upon MCT-1 knockdown, H1299 (2×10^6^) and A549 (1×10^6^) lung cancer cells were injected subcutaneously (s.c.) into the flank region at both sites of 6 week-old female BALB/c nude mice (BALB/cAnN-Foxnlnu/CrlNarl). All animal studies were conducted in accordance with the Animal Use Protocol approved by the National Health Research Institutes (NHRI-IACUC-098074-A). The tumor development was monitored every 3 or 4 days, and calculated by the formula: volume= 1/2 × L × W^2^, where L is the length and W is the width.

### Immunohistochemistry study

The tumor tissues were fixed in 10% formalin and embedded in paraffin. The 4 μm thick paraffin-embedded tissue sections were deparaffinized twice in xylene for 10 min and twice in ethanol for 2 min. The tumor sections were placed in 100 mM Tris-HCl (pH 6.0), 50 mM ethylenediaminetetraacetic acid (EDTA), heated at 92°C for 15 min and washed 3 times with PBS. After the endogenous peroxidase activity being blocked by 30% H_2_O_2_ for 5 min, samples were stained by the indicated Ab for 2 h, washed 3 times with PBS, incubated with LSAB2 kit/HRP System (DakoCytomation Denmark A/S, Glostrup, Denmark) and counterstained with hematoxylin. Images were observed by a Nikon Optiphot-2 Upright Microscope (Nikon Corporation, Tokyo, Japan) with a 20X objective lens, photographed by a Nikon DXM1200 CCD digital camera and analyzed by the Nikon ACT-1 imaging capture software under the same profile.

### MCT-1 and Shc mRNA expression levels in human cancers

The normalized cDNA samples of the TissueScan Lung Cancer Tissue qPCR Panel II, III and V (OriGene Technologies, Inc., Rockville, MD) were assayed the levels of MCT-1 and Shc gene activation in normal vs. cancerous lung tissues. The normalized cDNA samples from the TissueScan Breast Cancer Tissue qPCR Panel III and IV (OriGene Technologies, Inc.) were analyzed MCT-1 and Shc gene activation in normal vs. breast cancer tissues. The comprehensive pathology information for each sample is provided on the OriGene web site. The relative mRNA level of each target gene was analyzed by Q-RT-PCR and calculated by the formula: ΔC_T_ =C_t_ normal tissue group – C_t_ cancer group. The fold difference was calculated by the formula: 2^−ΔCT^.

### Statistics

The statistical analyses were assessed by Student's *t* test to compare the mean of control and experimental groups. The comparison between cancer and normal samples and the co-activation MCT-1 and Shc genes in human cancers were analyzed by Fisher's exact test (In-Silico Online; http://in-silico.net/). A *p* value<0.05 is considered to be statistically significant.

## Supplementary Figures and Tables




