# Supplementary material for: Targeting MCT-1 oncogene inhibits Shc pathway and xenograft tumorigenicity
Source: Oncotarget. 2012 Nov 9;3(11):1401–15. doi: 10.18632/oncotarget.688 (PMC3717801; doi:10.18632/oncotarget.688)
Supplement: Supplementary file 1 [file oncotarget-03-1401-s001.pdf]

Targeting MCT-1 oncogene inhibits Shc pathway and xenograft tumorigenicity - shih et al

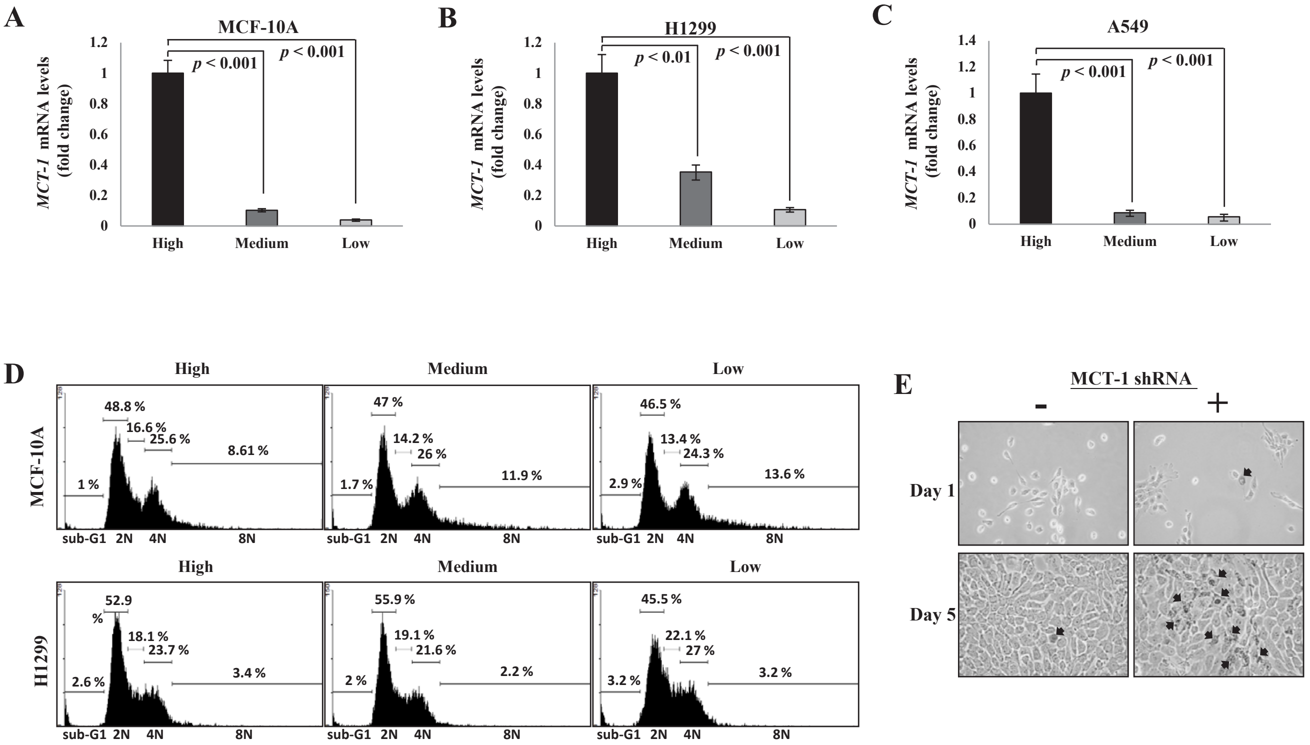

Supplementary Figure 1: MCT-1 gene silencing effects. MCF-10A, H1299, and A549 cells were transfected with the pGeneClip MCTS1 shRNA or empty vector. MCT-1 mRNA levels were examined in each cell line. The cellular RNA was isolated, reversely-transcribed into the complementary DNA (cDNA) and then analyzed by the quantitative real-time PCR (Q-RT-PCR). The control cells with a high-level of MCT-1 mRNA (High) were compared with the intermediately silencing cells (Medium) and the highly silencing cells (Low). (A) MCT-1 mRNA levels in MCF-10A cells reduced to 0.1-fold (Medium) and 0.03-fold (Low) over the control group (set as 1-fold). (B) MCT-1 mRNA levels in H1299 cells reduced to 0.35-fold (Medium) and 0.11-fold (Low) over the control sample (set as 1-fold). (C) MCT-1 mRNA levels in A549 cells reduced to 0.09-fold (Medium) and 0.06-fold (Low) over the control sample (set as 1-fold). (D) MCF-10A and H1299 cells were cultured in the regular media for 2 weeks and harvested for staining with propidium iodide (PI) followed by flow cytometry analysis. Knockdown of MCT-1 does not affect the cell cycle progression. (E) MCF-10A cell viability was analyzed by trypan blue exclusion assay after culturing in normal medium for 5 days. The incidence of spontaneous cell death (denoted by arrowheads) is induced by suppression of MCT-1.
