# Supplementary material for: Targeting MCT-1 oncogene inhibits Shc pathway and xenograft tumorigenicity
Source: Oncotarget. 2012 Nov 9;3(11):1401–15. doi: 10.18632/oncotarget.688 (PMC3717801; doi:10.18632/oncotarget.688)
Supplement: Supplementary file 2 [file oncotarget-03-1401-s002.pdf]

Supplementary Table 1.

Co-activation of MCT-1 and Shc genes in BCPs

	Shc (high)	Shc (low)
MCT-1 (high)	**52 (56.5%)**	16 (17.4%)
MCT-1 (low)	11 (12.0%)	**13 (14.1%)**

$p < 0.006$ (hyperactivation: low expression of MCT-1 and Shc genes)

Supplementary Table 1: Co-activation of MCT-1 and Shc genes in breast cancer patients (BCPs). The TissueScan Breast Cancer Tissue cDNA arrays Panel BCRT III and BCRT IV (OriGene Technologies, Inc.) were analyzed by Q-RT-PCR analysis. MCT-1 and Shc mRNA levels in each sample were normalized to β-actin mRNA level. MCT-1 transcripts with more than a 2-fold induction and Shc transcripts with more than a 1.5-fold increase over the mean of normal breast tissues were defined as the gene high-activation. Accordingly, 56.5% of the BCPs (n=92) highly induce MCT-1 and Shc genes, but only 14.1% of tumor biopsies exhibit low-expression of both genes. The statistical analysis used Fisher's exact test. The association of the MCT-1 and Shc gene induction in BCPs is statically significant (< 0.006).
